# The Effect of Patellofemoral Maltracking and Patella Type on Symptomatic Bipartite Patella

**DOI:** 10.7759/cureus.34076

**Published:** 2023-01-23

**Authors:** Musa Atay

**Affiliations:** 1 Radiology, Bagcilar Training and Research Hospital, Istanbul, TUR

**Keywords:** patella type, symptomatic, bipartite, trochlear dysplasia, patella

## Abstract

Purpose

The aim of this study is to investigate the relationship of trochlear dysplasia (TD) and patella type with bipartite patella (BP).

Materials and methods

A total of 5,081 knee MRIs taken in our institution were reviewed retrospectively. Patients with a history of knee surgery, previous or recent trauma, and rheumatologic involvement were excluded from the study. The MRIs of 49 patients with bipartite/multipartite patella were detected. Three patients were excluded: two patients had a tripartite variant, and one had multiple osseous dysplastic findings. Overall, 46 patients with BP were included in the study. BPs were classified as type I, II, and III. Patients were divided into symptomatic and asymptomatic groups according to the presence of edema within the bipartite fragment and adjacent patella. Patients were examined in terms of patella type, trochlear dysplasia, tuberosity-trochlear groove (TT-TG) difference, sulcus angle, and sulcus depth.

Results

There were 46 patients with BP (28 males and 18 females) (mean age: 33±9.5 years, range: 18-54). Thirty-eight bipartite fragments (82.6%) were type III and eight (17.4%) were type II. There was no type I BP. Seventeen (36.9%) were symptomatic, and 29 (63.1%) were asymptomatic. Seven of the type II (87.5%) and 10 of the type III (26.3%) bipartite fragments were symptomatic. The frequency and degree of trochlear dysplasia (p=0.007 and p=0.041, respectively) were found to be higher in symptomatic patients. The trochlear sulcus angle was higher (p=0.007) and the trochlear depth was lower (p=0.006) in the symptomatic group. No statistically significant difference was found (p=0.247) in terms of TT-TG difference. Type III and type IV patella were more common in the symptomatic group.

Conclusion

The current study shows that patellofemoral instability and patella type are associated with symptomatic BP. Patients with trochlear dysplasia, type II BP, and disproportionate patellar facet may have a significantly increased risk of symptomatic BP.

## Introduction

The primary and secondary ossification centers of the patella fuse around the age of 12. The absence of fusion is called bipartite patella (BP). In the case of multiple ossification centers, it is defined as multipartite patella. It is detected more frequently in men and is bilateral with a rate of 43%. It may cause anterior knee pain, especially after trauma, sports injury, or overuse [1,2]. Treatment is primarily conservative in symptomatic cases, and surgery may be preferred in cases unresponsive to conservative treatment [3]. There are few studies on symptomatic BP in the literature, and they reported that young age, thick cartilage surrounding the bipartite fragment, and medially and laterally tilted patellae are associated with symptomatic BP [4-6].

The aim of this study is to investigate the relationship of trochlear dysplasia (TD) and patella type with symptomatic BP. Our hypothesis is that patients with TD, type II BP, and disproportionate patellar facet may have a significantly increased risk of symptomatic BP.

## Materials and methods

**Figure 1:**
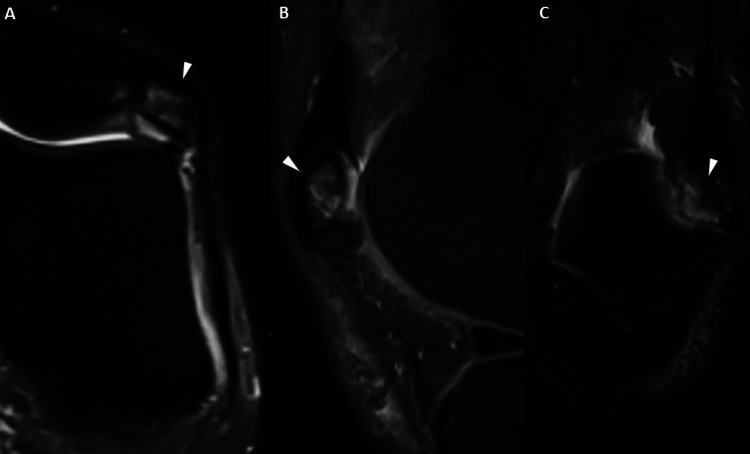
Symptomatic Bipartite Patella Axial PD (A), sagittal PD (B), and coronal (C) images show edema within the symptomatic fragment (arrowheads). PD: proton density

Between January 2022 and December 2022, 5,081 knee MRIs taken in our institution were reviewed in our study retrospectively. Patients with a history of knee surgery, previous or recent trauma, and rheumatologic involvement were excluded from the study. The MRIs of 49 patients with bipartite/multipartite patella were detected. Three patients were excluded: two had a tripartite variant, and one had multiple osseous dysplastic findings. A total of 46 patients with BP were included in the study. BP is classified as type I, II, and III. Patients were divided into symptomatic and asymptomatic groups according to the presence of edema within the bipartite fragment and adjacent patella and the absence of other major pathology that may cause pain in the knee [6]. Both groups were examined in terms of patella type, TD, tuberosity-trochlear groove (TT-TG) difference, sulcus angle, and sulcus depth (Figure 1).

MRI protocol

Unenhanced MRI of the knee was performed using a 1.5-T MRI unit with a dedicated knee coil in a 20-26 cm field of view and a 4-mm slice thickness with a 1-mm gap and a 320 × 256 matrix. The sequences used were sagittal T2 turbo spin-echo (TSE) (repetition time (TR): 2,300, echo time (TE): 82, flip angle (FA): 150), sagittal proton density (PD) with fat saturation (TR: 2,140, TE: 41, FA: 150), coronal PD with fat saturation (TR: 2,450, TE: 41, FA: 150), axial PD with fat saturation (TR: 3,330, TE: 47, FA: 150), and sagittal T1 SE (TR: 307, TE: 22, FA: 150).

Evaluation of bipartite patella and patella type

**Figure 2:**
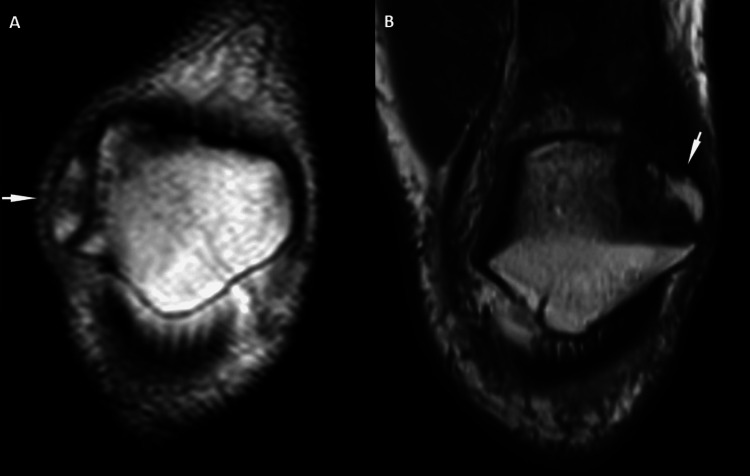
Bipartite Patella Types Coronal T1-weighted images show type II (A) and type III (B) bipartite patella (arrows).

According to the localization of the fragment, it was defined as type I if localized around the lower pole, type II if at the lateral side, and type III if at the superolateral pole (Figure 2) [7,8].

**Figure 3:**
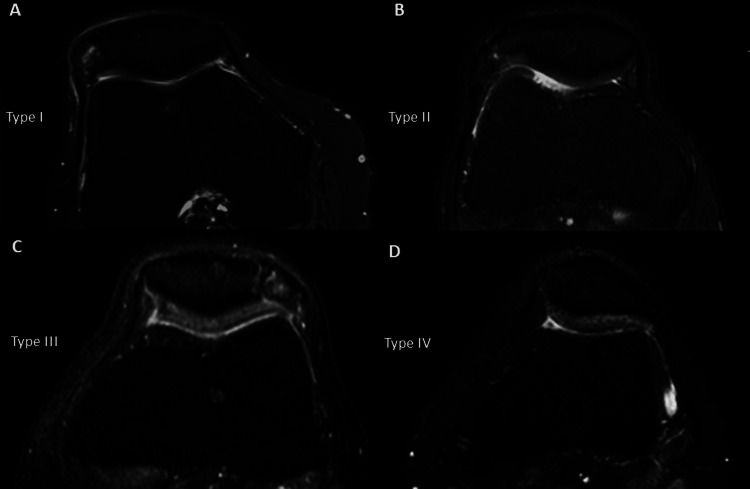
Patella Types Axial PD images show patella type I (A), type II (B), type III (C), and type IV (D). PD: proton density

The patella type was classified according to the Baumgartl classification: type I, both the medial and lateral facets are concave and of equal length; type II, the lateral facet is more prominent than the medial facet, and the medial facet is concave or straight; type III, there is a smaller and convex medial facet; and type IV, there is no medial facet (type IV is also called the “jockey cap”) (Figure 3) [9,10].

Evaluation of trochlear dysplasia

**Figure 4:**
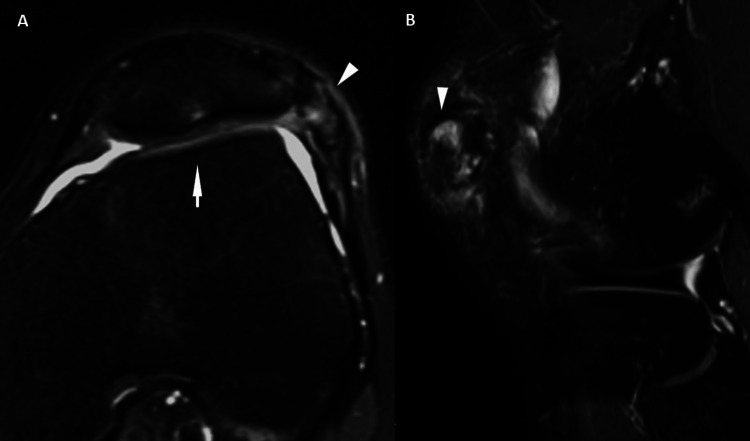
Trochlear Dysplasia and Bipartite Patella Axial PD and sagittal PD images show symptomatic bipartite fragment (A and B, arrowheads) and flattened trochlear groove consistent with trochlear dysplasia (A, arrows). PD: proton density

The evaluation and grading of TD were done according to the Dejour classification. The presence of shallow trochlea was defined as type A, flat trochlea as type B, asymmetric trochlear facets with hypoplastic medial condyle as type C, and type C plus cliff pattern as type D (Figure 4). TT-TG difference, trochlear sulcus angle, and trochlear depth were measured in terms of the degree of patellofemoral instability.TT-TG represents the transverse length between the trochlear groove and the tibial tuberosity on axial images.TT-TG distance measured less than 15 mm is considered normal. The sulcus angle is the angle between the medial and lateral facets. Values above 145° are evaluated in favor of TD. Trochlear depth measures the depth of the trochlear groove relative to the femoral condyles. Values below 3 mm are in favor of TD. These values are important parameters for patellofemoral instability associated with TD [11,12].

Statistical analysis

All statistical analyses were performed using Statistical Package for the Social Sciences (SPSS) version 20.0 (IBM SPSS Statistics, Armonk, NY, USA). In statistical analysis, the independent samples t-test was used to compare normally distributed variables within the groups. Pearson’s chi-squared test (x2) or Fisher’s exact test and Mann-Whitney U test, where appropriate, were used to compare the distribution of categorical data relative to each other. A one-way analysis of variance (ANOVA) test was used in terms of the age distribution of the groups. A p value of <0.05 was considered to show a statistically significant result.

## Results

The overall prevalence of BP was found to be 0.8%. There were 46 patients with BP (28 males and 18 females) in our study. The age range of the patients was between 18 and 54 years (average age: 33±9.5 years). Thirty-eight bipartite fragments (82.6%) were located superolateral to the patella (type III), and eight (17.4%) were located on the lateral side (type II). There was no type I BP. Seven of the type II bipartite fragments (87.5%) and 10 of the type III bipartite fragments (26.3%) were symptomatic, and a statistically significant difference was detected. Seventeen (36.9%) of all patients were symptomatic, and 29 (63.1%) were asymptomatic. There was no statistically significant difference between the symptomatic and asymptomatic groups in terms of age and male/female distribution (p>0.05). The demographic findings are shown in Table 1.

**Table 1:** Demographic Features Detailed demographic features of the patients with bipartite patella

	N=46
Age (years)	33±9.5
Range of age	18-54
Male	28 (60.8%)
Female	18 (39.2%)

TD was present in 18 (39.1%) of the patients. The frequency of TD (p=0.007) and the degree of TD (p=0.041) were found to be higher in symptomatic patients compared to asymptomatic patients, and there was a statistically significant difference. TD was detected in seven (7/29, 24.1%) patients of the asymptomatic group and 11 (11/17, 64.7%) patients of the symptomatic group. The distribution of TD according to the degree of dysplasia in the symptomatic and asymptomatic patients is shown in Table 2.

**Table 2:** Trochlear Dysplasia Types Distribution of patients according to trochlear dysplasia degrees TD: trochlear dysplasia

	Type A	Type B	Type C	Type D
Asymptomatic group (n=29)	5 (17.2%)	2 (6.8%)	-	-
Symptomatic group (n=17)	8 (47%)	2 (11.7%)	-	1 (5.9%)
TD total (n=18)	13	4		1

Although the TT-TG difference was slightly higher in the symptomatic group, no statistically significant difference was found between the groups (p=0.247). TT-TG difference, trochlear sulcus angle, and trochlear groove depth in symptomatic and asymptomatic patients are shown in Table 3.

**Table 3:** Trochlear Dysplasia Parameters Mean values of trochlear dysplasia parameters in the patient groups CI: confidence interval

	Asymptomatic group	Symptomatic group	p value
TT-TG difference	11.5±0.9 (95% CI: 9.57-13.43)	13.27±1.1 mm (95% CI: 10.87-15.66)	p=0.247
Sulcus angle	141.6°±2.2° (95% CI: 136.9-146.3)	143.2°±8.5° (95% CI: 122.2-158.3)	p=0.007
Trochlear depth	3.4±0.2 mm (95% CI: 3.03-3.7)	2.7±0.3 mm (95% CI: 2.1-3.3)	p=0.006

There was a statistically significant difference between the groups in terms of patella type, and type III and type IV patella types were more common in the symptomatic group. The proportional distribution in symptomatic and asymptomatic patients is shown in Table 4.

**Table 4:** Patella Types Patella types in the patient groups

	Type I	Type II	Type III	Type IV	Total
Asymptomatic group (n=29)	19 (65.5%)	7 (24.2%)	3 (10.3%)	-	29
Symptomatic group (n=17)	5 (29.4%)	4 (23.5%)	7 (41.2%)	1 (5.9%)	17
Total	24 (52.1%)	11 (23.9%)	10 (21.8%)	1 (2.2%)	46 (100%)

## Discussion

There are a few studies in the literature on BP. In the study of Duran et al., 18 patients were examined, and they stated that the defining feature of asymptomatic BP was thinner cartilage surrounding the fragment and synchondrosis [13]. O'Brien et al. found that the defining feature of asymptomatic BP was the intact and thin cartilage surrounding the synchondrosis and the fluid within the cleft [14]. To our knowledge, there are few studies on symptomatic BP in the literature. Ishikawa et al. stated that patients with symptomatic BP show significantly medially and laterally tilted patellae compared with the contralateral intact side [4]. In the study of Yeganeh et al., it was found that the cartilage diameter surrounding the fragment was higher in symptomatic cases than in the asymptomatic group [5]. Akdag et al. reported that the age of the patients in the symptomatic group was statistically lower than in the asymptomatic group [6].

There is no study in the literature investigating the relationship of symptomatic BP with patella type and TD. In our study, TD was present in 39.1% of patients, which is significantly higher than the prevalence of TD in the general population (approximately 10%) reported in the literature. In addition, the prevalence of TD in the symptomatic group (64.7%) was significantly higher than in the asymptomatic group (24.1%), and the rate of high-grade TD cases was also higher. We think that TD is associated with BP, especially in high-grade TD and symptomatic BP cases. Patellar instability caused by TD may have increased the instability of the bipartite fragment. Type II bipartite patella (87.5%) is more frequently symptomatic than type III bipartite patella (26.3%). This may be related to the fact that the laterally located (type II) fragment is relatively larger than the superolateral (type III) fragment and is more susceptible to perfusion defect or that the type II fragment is more affected by dynamic joint movement due to it being closer to the patellofemoral joint. Since type III and type IV patella are more common in the symptomatic group, the disproportionate facet ratio of the patella may be associated with the instability of the bipartite fragment.

The treatment of BP patients unresponsive to conservative treatment is surgical methods such as excision of the accessory fragment, lateral retinacular release, or open reduction and internal fixation [15]. TD, disproportionate facet ratio (type III and type IV), and type II BP may cause recurrent pain after surgery as they increase the risk of the accessory fragment becoming symptomatic. Therefore, these may be guiding findings in the selection of the surgical method. Additional intervention may be required during surgery, especially for TD.

There are limitations to our study. The first is the limited number of patients, the second is the retrospective nature of the study, and the third is that there is no radiological and clinical correlation.

## Conclusions

This study shows that patellofemoral instability and patella type are associated with symptomatic BP. The risk of symptomatic BP increases markedly, especially in the presence of type II BP, in the presence of a disproportionate patellar facet ratio, and in cases with high-grade TD. Clinicians should consider these possible pathologies when evaluating bipartite patella. These findings may guide surgical decisions in symptomatic cases.
